# Treatment of Combined Freckles with Chloasma Using Q-Switched 1064 nm Laser

**DOI:** 10.1155/2023/4081427

**Published:** 2023-06-26

**Authors:** Han Zheng, Gang Qiao, Yong Zhang

**Affiliations:** Department of Laser Center, Hangzhou Third People's Hospital, Hangzhou 310009, Zhejiang, China

## Abstract

**Objective:**

The present study observed the therapeutic effect and possible side effects of Q-switch 1064-nm laser with large-spot and low-energy technology in the treatment of patients with combined freckles and chloasma.

**Methods:**

A Q1064-nm laser with a large-spot diameter of 6–8 mm, energy level of 2.0–3.3 J/cm^2^, frequency of 10 Hz, and pulse width of 10 ns was employed for the treatment. Each patient underwent treatment 10–15 times, with an interval of 10–15 days each time. Facial care was administered before and after treatment; attention was paid to cleaning and moisturizing, avoiding light, and using sunscreens strictly. The therapeutic effects were observed and evaluated.

**Results:**

Freckles basically subsided (effective rate = 100%) and chloasma faded (effective rate = 39.4%). Furthermore, whitening and delicacy improvement were observed in the surrounding normal skin area. After laser treatment, confocal laser scanning microscopy revealed a large number of melanin particles in the upper part of the granular layer. Moreover, the amount of melanin in the middle and lower parts of the basal layer and spinous layer was significantly decreased. None of the patients developed postinflammatory pigmentation.

**Conclusion:**

In the treatment of freckles with chloasma, Q 1064-nm laser large-spot, low-energy technology not only removed freckles and faded chloasma but, most importantly, also reduced the incidence of postinflammatory pigmentation and improved patient satisfaction. This provided new methods and ideas for freckle laser treatment.

## 1. Introduction

Previously, Q755 nm or Q532 nm laser was used to treat freckled patients, and the curative effect was satisfactory [1]. However, there are still many patients with freckles and chloasma in clinic. If Q755 nm or Q532 nm laser was conducted to treat these patients, there would be serious side effects of pigmentation or deepening of chloasma after inflammation [2], which definitely make patients dissatisfied. Recently, the Q1064 nm laser: YAG laser technology with low energy and large spot has been widely used in the treatment of chloasma [3]. Therefore, we used Q1064 nm laser large spot, this low-energy technology, to treat this kind of freckles with chloasma and obtained certain curative effect and satisfaction. The details are reported as follows.

## 2. Data and Methods

### 2.1. Subjects

A total of 109 patients with chloasma-complicated freckles for skin type III∼IV between December 2010 and June 2013 were enrolled in the present study. All patients were females aged 27–53 years (average age = 41 years) with a disease course of 1–6 years. All 109 patients met the clinical diagnostic criteria for chloasma [4] (as follows).

Inclusion criteria include the following: (1) patients with patches on the face that had clear boundaries from light brown to dark brown; these were usually symmetrically distributed without inflammatory manifestations and scales and (2) patients without obvious subjective symptoms. The disease was seasonal (often severer in summer than in winter) and occurred more frequently in female subjects (primarily after puberty) than in male subjects.

Exclusion criteria include the following: (1) patients aged <18 years or >55 years; (2) patients in pregnancy or lactation; (3) patients with a history of drug allergy; (4) patients with serious endocrine diseases or other diseases (e.g., nevus of zygoma, vitiligo, Riehl melanosis, psoriasis, scarring constitution, diabetes, herpes simplex infection, and liver and cardiovascular diseases); (5) patients who underwent terminated treatment or received other therapies during treatment; (6) patients who had received other therapies three months before treatment; (7) patients who were unable to avoid light and/or use sunscreen after the operation; (8) patients with incomplete profiles affecting the judgment of the therapeutic effect; and (9) patients with pigmentation caused by other diseases.

### 2.2. Confocal Laser Scanning Microscopy (CLSM) Detection

The CLSM image feature analysis imaging system (vivascope1500, lucid Inc (US)) was used for low-power dermatoscope imaging and confocal scanning imaging of normal and typical skin lesions. A diode laser with a wavelength of 830 nm was employed. The objective lens magnification was 30x, and the CLSM spatial resolution was 5–10 mm horizontally and 3–5 mm vertically. The superficial epidermis and dermis tissues within 350 um could be detected.

Chloasma characteristics under CLSM: Melanocytes (MCs) and their dendrites may present with high refraction linear dendrite shadows, and pigment particles in keratinocytes may be characterized by dotted bright refraction scattering in the spinous and basal layers.

### 2.3. Therapeutic Methods

All patients were (1) informed of the treatment purpose, prognosis, and potential complications (hyperpigmentation, hypopigmentation, and inflammatory reaction) and signed informed consent; (2) photographed and archived before treatment; (3) treated using the Medlite C6 laser (Hoyaconbro company, USA) with a wavelength of 1,064 nm, large-spot diameter of 6–8 mm, low-energy level of 2.0–3.3 J/cm^2^, frequency of 10 Hz, pulse width of 10 ns, and treatment number of 10–15 (10–15 day interval each time); and (4) given facial care before and after treatment, with attention paid to cleaning and moisturizing, avoiding light, and strictly using sunscreens (SPF ≥ 30, PA++/+++).

### 2.4. Observation and Evaluation of the Therapeutic Effect

Chloasmata: (1) basically cured: more than 90% reduction of the chloasma area with naked-eye observation, and the skin color basically returned to normal; (2) excellent effect: 60%–89% reduction of the chloasma area under naked-eye observation, with no obvious boundaries between it and the surrounding normal skin; (3) effective: 30%–59% reduction of the chloasma area under naked-eye observation, with fuzzy boundaries between it and the surrounding normal skin; and (4) ineffective: less than 30% reduction of the chloasma area under naked-eye observation, with no significant change compared with before treatment. Total effective rate = basic cure rate + excellently effective rate [4].

Freckles: after treatment, the physician together with patient evaluated the improvement of freckles before and after treatment (1) Cured: the pigment decreased by 95%; (2) excellent: the pigment decreased by >50%; and (3) ineffective: the pigment decreased by <50%. Total effective rate = (cured cases + excellent cases)/treatment cases × 100% [5].

### 2.5. Statistical Processing

SPSS version 19.00 software was used to analyze the data. Analysis of variance was conducted to analyze the changes of indicators before and after treatment; *P* < 0.05 was statistically significant.

## 3. Results

**Figure 1:**
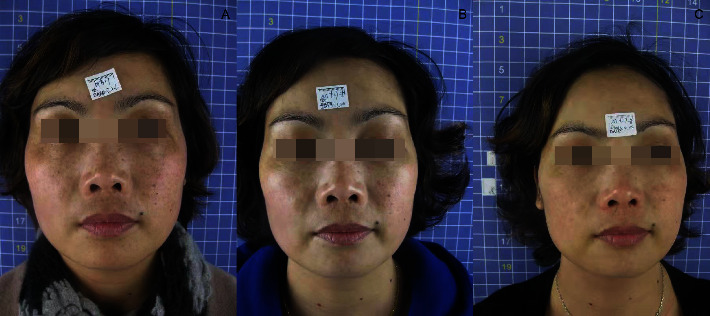
Comparison photo (a) before treatment; (b) after 5 treatments; and (c) one month after treatment.

**Figure 2:**
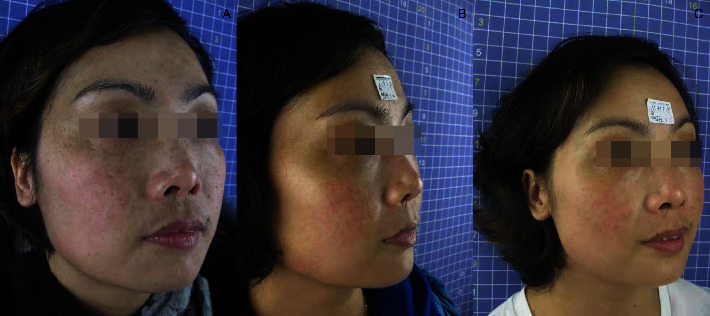
Comparison photo (a) right side before treatment; (b) after 5 treatments; and (c) 6 months after treatment.

**Figure 3:**
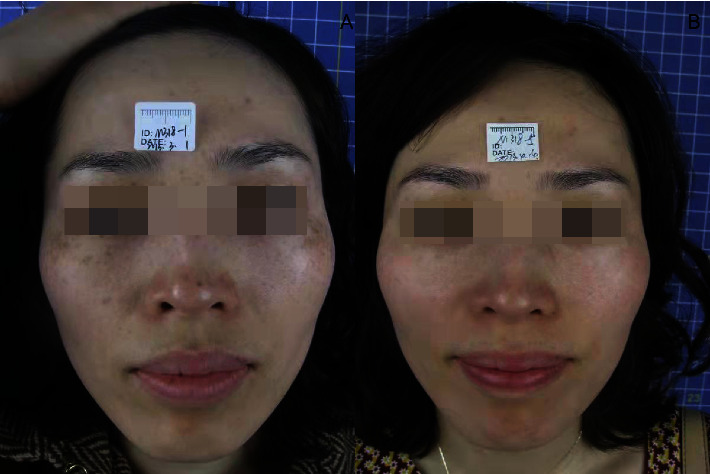
Comparison photo (a) before treatment and (b) after treatment.

**Figure 4:**
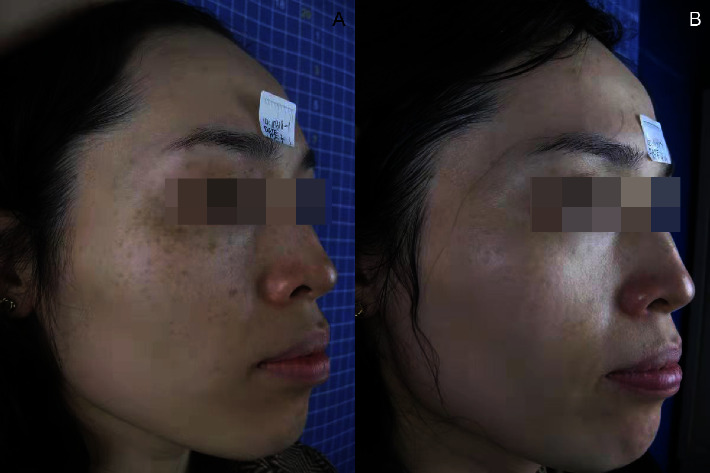
Comparison photo (a) before treatment and (b) after treatment.

After laser treatment, the freckles basically subsided (Table 1) and chloasma faded (Table 2). The efficacy of Q1064 laser for chloasma is significantly lower than that of Q1064 laser for freckles with *P* value of 0.013. The whitening and delicacy improvement were observed in the surrounding normal skin area. The comparisons of the states before, during, and after the treatment for the therapeutic effect are presented in Figures 1–4.

**Figure 5:**
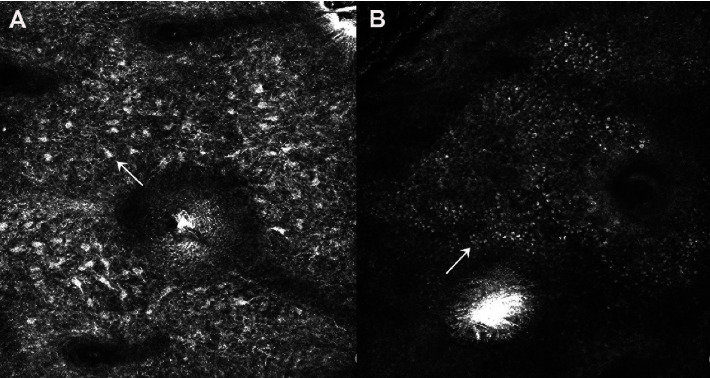
Effect comparison (A) before laser treatment with CLSM and (B) after laser treatment with CLSM.

In CLSM, the melanin particles were broken immediately, and the cell structure was blurred. After laser treatment, a large number of melanin particles could be seen in the upper part of the granular layer, and the amount of melanin in the middle and lower part of the basal layer and spinous layer decreased significantly (Figure 5).

**Figure 6:**
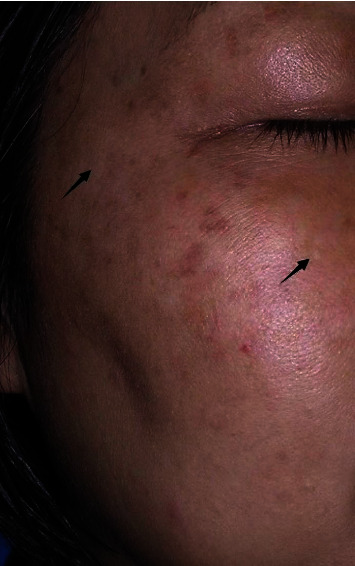
Clinical photos of patients who developed hypopigmentation.

Side effects: A total of 0 patients developed postinflammatory pigmentation, while 5 patients had temporary hypopigmentation spots (Figure 6). Hypopigmentation spots appear in the treatment area of chloasma and freckles, and no correlation with skin type was found. One patient had a family history of “vitiligo.” The other four cases were considered to be related to high-energy density parameters and unskillful treatment techniques. We externally use some ointments to help skin and pigment repair, such as asiaticoside ointment and xiletol ointment, which were rechecked once a month. After about three to six months. After about three to six months, the skin returned to normal, and no patients with persistent hypopigmentation were found.

Patients underwent a subsequent visit six months after treatment; A total of 3 patients had chloasma recurrence, and 0 patients had freckle recurrence.

## 4. Discussion

Freckles are brown punctate pigmentation spots commonly present on the face. They are obtained through autosomal dominant inheritance and often occur in exposed parts, especially the face. Freckles manifest as isolated (but not fused) small tan spots, which are aggravated after sun exposure. The optional laser treatment wavelengths are 755, 510, 532, and 694 nm. Usually, patients with obvious freckles can achieve ideal therapeutic effects; however, it is difficult to treat certain patients with freckles who have blurred skin lesions or in whom the condition is complicated with chloasma. The incidence of side effects of pigmentation or chloasma deepening after inflammation is very high; this has become a difficult treatment problem for doctors.

Melasma is a pigmented disease commonly present on the face. In the Asian population, melasma can affects 40% of women and 20% of men; this is especially true in areas with sufficient sunshine. At present, chloasma etiology and pathogenesis of are not fully understood. Most scholars believe that chloasma is caused by MC hyperfunction or increased activity and melanin formation acceleration caused by endocrine, ultraviolet (UV) radiation, pregnancy, microenvironment imbalance, genetics, oral contraceptives, and visceral diseases [6].

Melasma refers to pigment deposition after the activation of the melanocyte synthesis pathway caused by melanocytes becoming active. Melanin particles stimulate the formation of melanocytes in epidermis and hair follicles under UV radiation (especially 320–400 nm long-wave UV A (UVA)). UV radiation stimulates specific proteins and receptors (stem cell factor and c-kit protein), activates the binding of keratinocytes and melanocytes, releases epidermal cytokines (endothelial vasoconstrictor peptide), and binds to receptors on the melanocyte membrane. These cytokines can activate tyrosinase synthesis and increase melanin synthesis in melanosomes.

Chloasma is characterized by epidermis pigmentation with or without melanocyte infiltration. The amount of melanin in each layer of epidermis at skin lesions increases significantly, and the number and density of melanocytes in epidermis increase; there is an increase in solar elastic degeneration in the skin lesions, the number of melanosomes, and melanosome distribution in the keratinocytes.

There is also an increase in mitochondria, the Golgi apparatus, rough endoplasmic reticulum, and ribosomes in the cytoplasm of melanocytes in the skin lesion area. In summary, the occurrence of chloasma may be caused by the increase in the melanocyte number and the change in melanocyte synthase activity after sunlight irradiation.

For effective, long-term chloasma treatment, the synthesis of epidermal melanin needs to be controlled, the accumulation of melanin in melanocytes and keratinocytes reduced, the transport of melanin to keratinocytes increased, and the melanocytes in dermis mobilized.

At present, the main mechanisms for chloasma treatment include the following: (1) inhibiting tyrosinase using topical preparations to reduce melanin synthesis; (2) increasing melanin transport through chemical stripping; (3) destroying melanin using a pigment-selective laser; (4) remodeling skin with laser; and (5) partially destructing epidermal melanocytes and dermal melanocytes using a dot matrix laser. However, traditional treatment methods, such as preventing UV radiation, external decolorizing agent, and chemical stripping agent, have poor therapeutic effects, and the patient can easily relapse.

Use of the Q-switch 1064-nm laser, with an effective optical penetration depth of ≤1.7 mm, is an effective method for the treatment of skin pigmentation diseases [7]. It is often used in clinical practice to treat dermal pigmentation diseases, such as nevus of Ota and nevus fusco-cyanus of zygoma. The Q-switched 532-nm laser is mostly used to treat epidermal pigments, such as freckles, seborrheic keratoplaques, coffee spots, and freckle-like nevus.

The authors found that the condition of many patients with freckles in the laser clinic was complicated with chloasma. In the past, the treatment of freckles with small-spot, high-energy, and few times of Q532-nm laser usually caused damage to the surrounding skin tissue and basement membrane, resulting in chloasma deepening and aggravation as well as laser side effects of postinflammatory pigmentation.

Watanabe [1] revealed that laser may induce serious damage at high doses; this epidermal inflammatory response may lead to the release and oxidation of arachidonic acid, forming prostaglandins, and leukotrienes. These inflammatory mediators then stimulate MC in the epidermal layer and change its activity as well as the activity of related immune cells [8]. It can even lead to increased melanin synthesis and the formation of postinflammatory hyperpigmentation (PIH), i.e., the clinical “laser spot response” (also known as “light excitation”).

In recent years, scholars worldwide have employed the large-spot and low-energy (2.0–3.0 J/cm^2^) Q-switch Nd: YAG many times to treat superficial hyperpigmentation diseases, such as chloasma and postinflammatory pigmentation [2]. Therefore, the Q 1064-nm laser was employed in this study to treat patients with freckles and chloasma, so that the laser was partially absorbed by melanin, avoiding the aggravation of an inflammatory reaction caused by excessive melanin absorption of a short wavelength laser (400–800 nm).

In this study, the method of repeatedly used large-spot and low-energy laser could only exert selective light blasting for melanin particles in order to avoid or reduce the MC activation and inactivate or inhibit the MC function. This theory is called “subcellular selective photopyrolysis” [9].

When laser irradiates the skin at the lesion site, the melanin particles are selectively absorbed without damaging the surrounding normal tissue [10]. After absorbing energy, melanin particles expand rapidly and burst instantly to form small fragments; these are then swallowed by macrophages, excreted through lymphatic vessels or blood vessels, and transported to the stratum corneum for desquamation and excretion.

Chan et al. [11] also confirmed this by observing the three-dimensional structure of MC through a new three-dimensional surface-imaging technology. After laser treatment, only melanocytes were destroyed, and the number of MC dendrites decreased; however, the MC structure remained intact. In addition, multiple light explosions of melanin particles could make melanin particles smaller and more conducive to being swallowed and discharged. This “subcellular selective photolysis” can also minimize the damage of laser to normal skin tissue and the basement membrane as well as avoid the occurrence of PIH.

The limitation of this article is the lack of long-term follow-up on the recurrence of chloasma with freckles, and we will do more work within this area in the further study.

Although the laser fades chloasma, it also eliminates freckles on chloasma. This method is safer than the traditional laser treatment of freckles, prevents the occurrence of pigmentation side effects [12] and improves patients satisfaction. It also provides new methods and ideas for laser treatment of freckles.

## Figures and Tables

**Table 1:** Statistics of therapeutic effect of freckles.

Total	Cured	Excellent	Ineffective	Total effective rate (%)
109	97	12	0	100

**Table 2:** Statistics of therapeutic effect of chloasma.

Total	Basically cured	Excellent	Effective	Ineffective	Total effective rate (%)
109	18	25	59	7	39.4

## Data Availability

The datasets used and/or analyzed during the current study are available from the corresponding author on reasonable request.
